# From Academic Resilience to Academic Burnout among International University Students during the Post-COVID-19 New Normal: An Empirical Study in Taiwan

**DOI:** 10.3390/bs13030206

**Published:** 2023-02-27

**Authors:** Thanh Xuan Tran, Thi Thuy Tien Vo, Chen Ho

**Affiliations:** 1 International Business and Trade Program, Ming Chuan University, Taipei 111, Taiwan; 2 Faculty of Odonto-Stomatology, Hong Bang International University, Ho Chi Minh City 72500, Vietnam

**Keywords:** academic burnout, academic resilience, new normal

## Abstract

(1) Background: In the context of the COVID-19 pandemic, it is imperative for higher education institutions to understand the socio-psychological issues of international students, a potentially vulnerable population on campuses, to assist them in pursuing their academic path while maintaining their psychological well-being. The objectives of this study were to determine the prevalence of academic burnout among international university students in Taiwan during the new normal and to explore the protective role of academic resilience. (2) Methods: Three hundred and eighty-three international university students in Taiwan were recruited and surveyed via the online self-administered questionnaire during the Fall semester of the 2022–2023 academic year. The data of sociodemographic characteristics, academic burnout, and academic resilience were collected and analyzed. (3) Results: The overall prevalence of high academic burnout was 12.01%. The majority of participants perceived significant depression and anxiety (detrimental factors) but moderate to high perception of academics and relationships (protective factors). There were significant relationships between resilience components and burnout symptoms. (4) Conclusions: Resilience may help to reduce burnout among international university students during the post-COVID-19 new normal, thereby protecting their mental health.

## 1. Introduction

International students are important to achieve the goal of Taiwan becoming a bilingual nation by 2030. There have been many attractive subsidies and scholarships as well as national policies to encourage the enrollment of international students in Taiwan [1,2]. The influx of international students to Taiwan’s higher education institutions (HEIs) increased from 45,413 in 2010 to 128,157 in 2019 [3]. The increasing number of international students inevitably brought along an increase in challenges for both the host country and the foreign students. During the COVID-19 pandemic, HEIs worldwide faced unprecedented challenges [4], including Taiwan’s university sector. University students dealt with an unconventional reorganization of learning activities coupled with a wide range of psychological, financial, and relational issues [5,6,7]. Studies focusing on the psychological consequences of the pandemic among university students have reported negative mental health impacts such as stress, depression, and anxiety [8,9,10]. A few investigations conducted in Taiwan during the COVID-19 crisis showed similar results. Lin et al. (2021) found that the pandemic drastically affected Taiwanese students’ financial, educational, and social conditions, which were significantly associated with mental health outcomes (i.e., loneliness, anxiety, and depression) [11]. Ahorsu et al. (2021) indicated that international students exhibited different COVID-19-related challenges and had higher anxiety as compared with Taiwanese students [12]. This is likely due to the fact that international students may experience more difficulties due to language barriers, cultural changes, financial hardships, homesickness, and loss of social support [13,14,15]. Therefore, it is imperative to take the initiative to reach out to international students in Taiwan’s colleges and universities to understand their perspectives and experiences, thereby developing efficient strategies to support an inclusive academic environment.

The term new normal was emerged during the financial crisis of 2007–2008 to describe the significant economic, cultural, and social transformations that result in individual and social unrests [16]. Since the world first went into the COVID-19 outbreak in 2020, this term has been coined again to emphasize how everything has drastically changed. Although the era of new habits due to the pandemic has been expected to maintain the learning activities as the pre-pandemic period, difficulties adapting to this new normal still remain. Moreover, it is possible that we would experience similar health crises in the future.

Academic burnout, a common problem that afflicts students around the world in terms of mental health and academic achievement., is a three-dimensional syndrome of emotional exhaustion, cynicism, and reduced efficacy [17,18]. The transition to university and the challenges in higher education can increase susceptibility to the symptoms of academic burnout in students. Increasing evidence has indicated the significant prevalence and degree of burnout symptoms in university students since the COVID-19 outbreak [19,20,21]. Nonetheless, the literature has mainly mapped the trends related to local students and failed to highlight those related to international students. Being a minority on campuses, the needs of international students may be neglected, leaving them feeling disappointed and frustrated. As a result, they are at a higher risk for academic burnout, resulting in negative effects on their emotions, their engagement in the learning process, and their mental well-being [19,22]. Indeed, a cross-sectional survey of Indian students at a Russian medical university found that 40% of respondents showing a deterioration in psycho-emotional well-being and a high level of burnout due to the pandemic [23]. There seems to be no evidence of such studies, however, being done with international students in Taiwan.

In the wake of the COVID-19 pandemic, research on human resilience may provide helpful implications for children and youth to prepare and respond to a crisis [24]. Resilience is defined as the process and outcome of successfully adapting to difficult or challenging life experiences, especially through mental, emotional, and behavioral flexibility and adjustment to external and internal demands [25]. As university students inevitably face competing academic, social, and financial pressures and setbacks, resilience plays an important role for them to better withstand these challenges [26]. Resilient students have a more positive attitude and more engagement in learning when facing with adverse academic situations. They also have a better ability to improve their academic adjustment and performance [27]. It was found that academic resilience would shape students who are affected by the COVID-19, and its effect on the current situation would ultimately give students a conclusion about the condition where they are living [28]. Thus, academic resilience may serve as a protective factor for academic performance and act as a way for students to cope with academic burnout [29].

To navigate the way through unforeseen and fluctuating circumstances, HEIs need to identify the capacity of their stakeholders, especially students, to address issues related to potential socio-psychological damages not only during the crisis but also in the new normal after that. The present research therefore aimed to investigate the psychological experiences of international students studying at HEIs in Taiwan, where they comprised approximately 7.8% of university student population. In particular, our objectives were to examine the prevalence of academic burnout among international university students in Taiwan during the post-COVID-19 new normal and to highlight the positive effects of academic resilience on this mental health issue.

**Figure 1:**
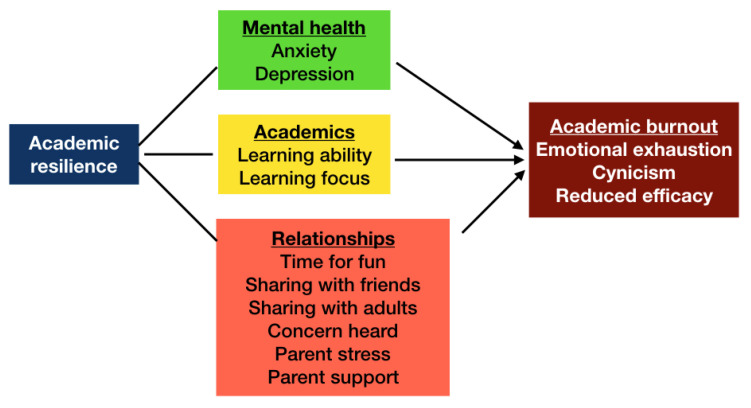
Conceptual framework that hypothesizes a relationship between academic resilience and academic burnout.

Considering the job demand-resources (JD-R) model of burnout, which is proven as an appropriate framework to predict student burnout in higher education [30], this study assessed the moderating model of resilience, which hypothesized that higher levels of academic resilience associate with lower scores of academic burnout among international students. Psychological and emotional demands are the core components of the JD-R model of burnout, which are linked with strain and exhaustion [30]. In higher education, anxiety and depression have been proven to cause harmful emotional load [31,32]. Among resources, perceived possibility of personal growth and development, control, information, feedback, and support of lecturers are included as important components of the JD-R model [30]. Moreover, the literature has indicated that social support (environmental resources) is a crucial factor that may alleviate the level of academic burnout, as it is easier for students to seek help when faced with adversity [33]. Our conceptual framework that hypothesized the relationship between academic resilience and academic burnout is illustrated in Figure 1. Our findings suggest that recommendations for HEIs include initiatives to help international students enhance their learning ability and learning focus while maintaining their family support and social network.

## 2. Materials and Methods

### 2.1. Study Design and Participants

This study was a cross-sectional survey of international students from colleges and universities across Taiwan. To be eligible, participants were international students who had lived and studied in Taiwan throughout the COVID-19 outbreak from May 2021 to August 2021 until the study time point. According to Alam et al. (2021), we computed the sample size based on the population size of 92,963 students. A minimum sample size of 383 foreign university students was required [3,5]. The survey was shared among the target population through the convenience sampling method until the recommended sample size was reached.

### 2.2. Data Collection

The data were collected during the fall semester of the 2022–2023 academic year. The survey instrument was delivered as an online questionnaire using Google Form to be easily shared and accessed electronically. In addition, the participants were encouraged to further share the survey link to their friends that they considered suitable for this survey. After clicking the link, the participants were fully informed about the purpose of this study, and the confidentiality of their information was warranted. When the participants voluntarily agreed to participate in the research, they anonymously completed the self-administered questionnaire for no financial incentive. The participants were free to discontinue their participation at any time.

### 2.3. Survey Instrument

The survey consisted of three parts, including sociodemographic characteristics, academic burnout, and academic resilience. To capture the academically psychological experiences, the Maslach Burnout Inventory (MBI)-Student Survey (MBI-SS) and Student Resilience Survey (SRS) were used.

Maslach Burnout Inventory was introduced by Maslach and Jackson in 1981 for measuring three theoretical dimensions of burnout syndrome. An adaptation of the MBI termed MBI-SS was developed for college students by Schaufeli and colleagues in 2002 [17]. MBI-SS consists of 15 items that constitute three domains, including exhaustion (EX; 5 items), cynicism (CY; 4 items), and efficacy (EF; 6 items). All items are written in the form of statements about personal feelings or attitudes, and they are scored on a 7-point frequency rating scale ranging from 0 (Never) to 6 (Always). It was reported that MBI-SS is a valid and reliable tool to assess college students’ burnout status [34]. In this study, the Cronbach alpha’s coefficients of EX, CY, and EF were all higher than 0.9, indicative of excellent reliability of the instrument.

Authentic Connections (AC) is a team of leading scientists, clinicians, and consultants committed to help school measure, track, and improve student well-being and resilience. In 2020, Student Resilience Survey was designed to be a short survey by AC to help schools assess the impact of disruptions resulting from the COVID-19 pandemic on students’ well-being and mental health. SRS measures two components of mental health, depression and anxiety. In addition, it evaluates two essential components of resilience during the COVID-19, academics and relationships. The former domain includes learning ability and learning focus, whereas the latter consists of time for fun, sharing with friends, sharing with adults, concern heard, parental stress, and parental support. SRS is a mixed-methods survey that includes both quantitative statements and open-ended questions [35]. To simplify our survey instrument, we transformed the questions of SRS into equivalent quantitative statements. All of quantitative items were measured by asking participants to report how they experience the issue described in every statement on a 5-point frequency rating scale ranging from 0 (Never) to 4 (Very Often). In this study, the Cronbach alpha’s coefficient values for mental health, academics, and relationships were 0.79, 0.62, and 0.60, respectively, indicating a relatively acceptable reliability of the simplified SRS. The rather low values of academics (0.62) and relationships (0.60) may be due to the heterogeneity of the items assessed. This fact was taken account into the limitations of the present study.

### 2.4. Data Analysis

Data were statistically analyzed by using the GraphPad Prism^TM^ software (GraphPad, San Diego, CA, USA). Our primary analysis involved the descriptive summary of the socio-demographic characteristics of the participants, the prevalence of academic burnout and resilience. The numerical variables were presented as mean (M) ± standard deviation (SD), while the categorical variables were expressed as number and percentage. For univariate analyses, Mann Whitney-U test and Kruskal Wallis test were performed to compare the mean values of academic burnout and resilience between two or more groups, respectively. Next, Pearson’s correlation coefficient was used to determine the relationships either between the variables of academic burnout and resilience. Finally, regression analyses were done separately for each of three dimensions of academic burnout as an outcome. All the sociodemographic variables and academic resilience components statistically correlated with the outcomes in previous tests were included in the multiple regression analysis. Values *p* < 0.05 were considered statistically significant.

## 3. Results

### 3.1. Description of Study Sample

A total of 383 international university students in Taiwan (overall response rate 98.7%) completed the questionnaire online between August 2022 and November 2022. By age, the average was 23 ± 5.045 years, with a range between 18 and 48 years old. The descriptive statistics of other sociodemographic characteristics are shown in Table 1. Of 383 respondents in total, 64.5% were female and 95.3% were single. The distribution of grades was relatively even, with 15.14% in the first year of study, 18.28% in the second year, 23.5% in the third year, 23.24% in the fourth year, and 19.84% were postgraduates and others. A business major accounted for more than half of participants (51.44%). Although there were representatives from all the continents, most of them were from Asia (71.54%). Regarding living conditions, approximately one-third of respondents were living alone (34.73%). For further inferential statistics, we decided to dichotomize the variables “marital status” and “continent” into their highest frequencies and less than highest frequencies because of the extremely skewed distributions. Similarly, the variable “year of study” was also reclassified into two groups of undergraduates and postgraduates.

### 3.2. Prevalence of Academic Burnout

As shown in Table 2, approximately 43% (*n* = 164) and 57% (*n* = 220) of participants were classified as having high EX and high CY, respectively, while more than 27% (*n* = 106) of them presented low EF. The overall prevalence of high academic burnout (high EX, high CY, and low EF) was 12.01% (*n* = 46).

To further determine possible predictors of academic burnout among participants, a series of univariate analyses was run with sociodemographic characteristics as independent variables and each burnout dimension as a dependent variable. As shown in Table 3, the mean scores of EX were significantly higher in undergraduates as compared with postgraduates (13.85 vs. 9.16; *p* < 0.001) and in non-Asian as compared with Asian (14.91 vs. 12.54; *p* = 0.025). For the CY subscale, the mean scores were significantly higher in single participants as compared with non-single ones (8.94 vs. 5.44; *p* = 0.013) and in undergraduates as compared with postgraduates (9.62 vs. 5.37; *p* < 0.001). For the EF subscale, the mean scores were significantly lower in undergraduates as compared with postgraduates (20.27 vs. 23.34; *p* = 0.007) and in Asian as compared with non-Asian (19.82 vs. 23.54; *p* = 0.001). Significant differences in the major of study were emerged on EX (*p* = 0.001) and CY (*p* < 0.001) but not EF (*p* = 0.194). Neither sex nor living conditions affected three dimensions of burnout. However, the multivariate analyses (Table 4) showed that only year of study and continent were significantly associated with burnout symptoms. Postgraduates had lower EX and CY scores but higher EF scores than undergraduates, whereas non-Asian students had higher scores in all three burnout dimensions than their Asian counterparts. These results suggest that international students who were undergraduates and originated from non-Asia nations were more susceptible to burnout syndrome.

### 3.3. Perception of Academic Resilience

The students were classified as having low, moderate, and high levels for each component of SRS was based on the cutoffs at average ± 1.5 SDs. As shown in Table 5, only 26.37% (*n* = 101) of participants considered themselves as having low depression and anxiety. However, the majority of respondents presented moderate-to-high resilience in terms of academics and relationships (protective factors).

Again, to further determine possible predictors of academic resilience among participants, a series of univariate analyses was run with sociodemographic characteristics as independent variables and each SRS component as a dependent variable. As shown in Table 6, males had significantly higher mean scores of mental health than female did (5.07 vs. 4.63; *p* = 0.041). Single participants showed significantly higher mean scores of mental health (4.83 vs. 3.78; *p* = 0.024) and lower mean scores of relationships (12.57 vs. 14.83; *p* = 0.045) as compared with non-single ones. Undergraduates exhibited significantly higher mean scores of mental health (4.91 vs. 4.29; *p* = 0.009) and lower mean scores of academics (4.09 vs. 5.07; *p* < 0.001) and relationships (12.42 vs. 13.70; *p* = 0.012). Additionally, major of study and living conditions were significantly associated with academics and relationships while there were no significant differences between participant from different continents on any component. However, the multivariate analyses (Table 7) only showed that female (compared to male) had lower scores of mental health, non-single participants (compared to single participants) showed higher scores of relationships, and not living alone (compared to living alone) exhibited lower scores of academics and relationships. These results suggest that international students who were female were more susceptible to anxiety and depression, while those who were single and living alone considered themselves more resilient in terms of academics and relationships.

### 3.4. The Relationship between Academic Resilience and Academic Burnout

Pearson’s correlation analyses were carried out to explore the relationships between three academic burnout dimensions and three academic resilience components. As shown in Table 8, although academics and relationships were negatively correlated with the CY subscale and positively correlated with the EF subscale, there was no correlation between the academic resilience components and the EX subscale.

After determining significant correlations between each dimension of academic burnout with the sociodemographic characteristics and academic resilience components, multivariable linear regression was performed to identify the predictors of the EX, CY, and EF subscales. In addition, age and self-reported financial status were also included into the models. As shown in Table 9, mental health remained having no significant effect on burnout symptoms. However, after controlling possible sociodemographic confounders, academics and relationships were revealed to be significant predictors of EF but not of CY. In particular, higher perceived resilience in terms of academics and relationships was associated with a higher personal efficacy and development.

## 4. Discussion

The COVID-19 outbreak and accompanying limitations have wreaked havoc on the education industry. HEIs are inevitably face a catastrophe with the emergence of the COVID-19 pandemic [4]. In Taiwan, university campuses initially maintained normal operation, where students attended their classes as scheduled but under strict regulations to ensure the safety of students and faculties. Shortly after, online instruction was integrated into face-to-face classes in response to the sporadic spread of the virus, and further shifted to full online teaching due to the outbreak of infection. Recently, university campuses have been reopening, and many students have been heading back to in-person classes after a period of social distancing. This has returned students to the new normal where they change their attitudes and behaviors to continue their engagement in learning activities while preventing the spread of infection [36,37]. Although the new normal is expected to maintain the learning activities as normal, the campus life may still become more stressful than usual for international students, a potentially vulnerable group. Thus, not to be missed in the unforeseen and fluctuating circumstance of the COVID-19 and future crises is finding out how this at-risk population psychologically experience the campus life during the post-pandemic new normal.

Burnout is an emerging mental health issue in the contemporary society, which affects humans in various sectors [38]. There is increasing evidence to indicate the academic burnout experienced by students, in which students in higher education are at a high risk [30]. A recent meta-analysis estimates that the pooled prevalence of burnout among university students is 12.1% [39]. In the context of the COVID-19 crisis, research that investigated foreign medical students in Eastern Europe reported that 39.1% of respondents presented psycho-emotional issues due to the pandemic, including depression (12.9%), exhaustion (19.9%), loneliness (25.2%), nervousness (20.0%), and anger (23.3%). Moreover, up to 97.4% and 90.8% of them were positive for emotional exhaustion and cynicism [23]. An online-based cross-sectional study among undergraduate students at Jordan showed that 6.6% of participants were found to have symptoms of burnout during distance learning period [40]. In line with previous findings, the present study indicated that the overall prevalence of high academic burnout among international university students during the new normal in Taiwan is as high as 12.01%. This significant prevalence of high academic burnout may be attributed to the lack of support available to foreign students when being in abroad, especially during a crisis. For this reason, there should be a surge in the need for mental health services and supportive counseling dedicated to international students in HEIs.

Academic resilience is the ability of a student to overcome academic adversity and challenges, from institutional to financial to relationship problems, which can affect their academic performance [26]. Thus, academic resilience helps students manage the challenges associated with the process of academic achievement, correlates with academic success, and acts as a way for students to cope with academic burnout [27,29]. A case study of Taiwanese university students during the COVID-19 pandemic indicated that they were moderately resilient across all age groups. While facing the crisis, students exhibited their capacity to withstand and operate with the given resources to build the resilient environment for their well-being [41]. Since the COVID-19 outbreak in 2020, the prevalence and exacerbation of mental health issues, particularly those related to depression and anxiety, have increasing in university students [42,43]. Similarly, many of international university students who were analyzed in this study reported noticeable depression and anxiety during the new normal. However, the majority of them appeared to well adapt to the new normal by showing moderate and high resilience in terms of academics and relationships. Therefore, our results suggested that international university students considered themselves as highly capable of overcoming obstacles and hardships imposed by the pandemic. In addition, multivariate analyses of sociodemographic variables further indicated that the students who showed the high rates of resilience presented the profiles as being female, single, and living alone.

Research conducted since the COVID-19 pandemic has indicated that resilience is a positive contributor to psychological health and mental well-being [44]. In this study, the Pearson’s correlation analyses revealed that resilience components had a role in the prevention of burnout symptoms. A recent meta-analysis reported a significant association between burnout with either depression or anxiety [45]. However, our results found no correlation between these two mental health issues with burnout dimensions among international university students during the new normal. This might be due to the short duration of the COVID-19 closure, which lasted approximately three months in Taiwan. In fact, it was demonstrated that the post-traumatic effect of a confinement is dependent the duration of that confinement [46]. In contrast, our study found that international university students who perceived positive academics and relationships presented lower cynicism and higher efficacy. It can be supposed that, due to the COVID-19 pandemic, international university students may have experienced many changes in their learning activities, relationships, and lifestyles, resulting in more adversities in their abroad lives. Thus, favorable academics and relationships may serve as protective factors that help them feel more ease to overcome the difficulties by maintaining learning ability, learning focus, fun, and support. Nonetheless, after controlling the possible confounding factors (including age, sex, marital status, year of study, continent, living conditions, and financial status), three components of academic resilience significantly associated with neither EX nor CY, whereas academics and relationships were significant predictors of EF. Our findings are reasonable as perceived possibility of personal growth and development, control, information, feedback, and support of lecturers are included as critical resources in the JD-R model of burnout development [30]. Moreover, social support as environmental resources is also an important element that may reduce the level of academic burnout [33]. Taken together, our results suggested that during challenging time such as the COVID-19 pandemic and the subsequent new normal, improvement of academics and relationships may help international university students possess higher levels of resilience, mitigating burnout syndrome and enhancing mental well-being of students. Therefore, HEIs should aim at increasing resilience for their students, particularly foreign students, to assist them prepare and respond more healthily and effectively to a crisis.

There are several limitations in this study. First, since the COVID-19 pandemic hit different areas in Taiwan at different levels of severity, it would be helpful to recruit a larger sample across various institutions using the stratified nationwide sampling to reflect the diversity of target population. Second, this was a cross-sectional study without comparison to data sets prior to the COVID-19 pandemic due to the paucity of relevant studies. Therefore, further longitudinal research and comparative analyses are required to verify the predictive power of academic resilience on the occurrence and development of the academic burnout. Third, this study used the online self-administered survey for collecting data, which may have given biased information and restricted access. Moreover, the internal consistency values for the modified SRS found in the present study were somewhat questionable. Future studies should employ a variety of measures and approaches to withdraw more comprehensive and powerful conclusions.

## 5. Conclusions

The pandemic has caused sudden changes in socioeconomic conditions that have permanently reshaped the learning environment. Adjusting to this new normal has been especially challenging for international students. Our study fills a gap in post-COVID-19 research on resilience in HEIs and advances scholarly understanding of socio-psychological issues faced by international students. Our findings indicate a significant prevalence of high academic burnout among international university students during the new normal and suggest that cultivating academic resilience is crucial to promote their emotional and mental well-being. Academic resilience has promising potential to mitigate the symptoms of academic burnout, which could inform new HEI policies to provide avenues for both local and international students to thrive and adapt during unexpected crises.

## Figures and Tables

**Table 1:** Sociodemographic characteristics of respondents (N = 383).

Sociodemographic Characteristic		N = 383	
		*n*	(%)
Sex	Female	247	(64.50)
	Male	136	(35.50)
Marital status	Single	365	(95.30)
	Married	15	(3.92)
	Others	3	(0.78)
Year of study	First year	58	(15.14)
	Second year	70	(18.28)
	Third year	90	(23.50)
	Fourth year	89	(23.24)
	Master	31	(8.09)
	Ph.D.	34	(8.88)
	Others	11	(2.87)
Major of study	Education	20	(5.22)
	Science/Medicine/Biology	40	(10.44)
	Engineering	16	(4.18)
	Agricultural	2	(0.52)
	Business	197	(51.44)
	Social science/Law	33	(8.62)
	Language/Arts/Communication	75	(19.58)
Continent	Asia	274	(71.54)
	Africa	11	(2.87)
	North America	25	(6.53)
	South America	17	(4.44)
	Europe	21	(5.48)
	Australia/Oceania	14	(3.66)
	Others	21	(5.48)
Living conditions	Alone	133	(34.73)
	With friends	186	(48.56)
	With relatives	37	(9.66)
	Others	27	(7.05)

**Table 2:** Prevalence of academic burnout among respondents (N = 383).

Academic Burnout Subscale	N = 383	
	*n*	%
Exhaustion (EX)		
Low (0–9)	122	(31.85)
Moderate (10–14)	97	(25.33)
High (>14)	164	(42.82)
Cynicism (CY)		
Low (0–1)	52	(13.58)
Moderate (2–6)	111	(29.00)
High (>6)	220	(57.42)
Efficacy (EF)		
Low (>27)	106	(27.68)
Moderate (23–27)	80	(20.89)
High (<23)	197	(51.43)

**Table 3:** Academic burnout scores across sociodemographic characteristics among respondents (N = 383).

Sociodemographic Characteristic	Exhaustion (EX)		Cynicism (CY)		Efficacy (EF)	
	M (SD)	*p*-Value	M (SD)	*p*-Value	M (SD)	*p*-Value
Sex		0.619 ^1^		0.265 ^1^		0.077 ^1^
Male	12.91 (8.80)		8.32 (6.99)		21.92 (9.46)	
Female	13.38 (8.26)		9.02 (6.86)		20.31 (9.23)	
Marital status		0.288 ^1^		0.013 ^1^		0.077 ^1^
Single	13.32 (8.47)		8.94 (6.88)		20.81 (9.32)	
Non-single	11.06 (7.82)		5.44 (6.77)		22.39 (9.73)	
Year of study		<0.001 ^1^		<0.001 ^1^		0.007 ^1^
Undergraduate	13.85 (8.34)		9.62 (6.86)		20.27 (9.17)	
Postgraduate	9.16 (7.97)		5.37 (5.54)		23.34 (9.61)	
Major of study		0.001 ^2^		<0.001 ^2^		0.194 ^2^
Education	16.15 (8.98)		11.35 (8.00)		22.10 (9.93)	
Science/Medicine/Biology	7.90 (7.65)		3.90 (4.59)		23.03 (10.30)	
Engineering	12.38 (8.94)		5.44 (4.43)		21.81 (9.33)	
Agricultural	17.00 (11.31)		14.00 (8.49)		9.00 (0.00)	
Business	13.99 (8.35)		9.84 (7.10)		20.45 (9.09)	
Social science/Law	13.42 (8.53)		8.36 (5.73)		22.27 (8.98)	
Language/Arts/ Communication	13.19 (7.96)		8.65 (6.69)		20.07 (9.36)	
Continent		0.025 ^1^		0.071 ^1^		0.001 ^1^
Asia	12.54 (7.97)		8.30 (6.57)		19.82 (9.46)	
Non-Asia	14.91 (9.37)		9.96 (7.59)		23.54 (8.47)	
Living conditions		0.388 ^2^		0.288 ^2^		0.151 ^2^
Alone	13.65 (8.31)		9.42 (6.59)		22.44 (8.38)	
With friends	12.60 (8.43)		8.25 (6.94)		19.92 (9.44)	
With relatives	13.19 (8.67)		9.46 (7.57)		19.95 (11.20)	
Others	15.26 (9.01)		8.30 (7.26)		21.11 (9.69)	

^1^ Mann–Whitney *U* test. ^2^ Kruskal–Wallis test.

**Table 4:** Multivariate analyses on sociodemographic characteristics with academic burnout.

Sociodemographic Characteristic	The Beta Coefficient (β)		
	Exhaustion (EX)	Cynicism (CY)	Efficacy (EF)
Sex	0.680	0.906	−0.082
Marital status	0.529	−0.480	0.600
Year of study	−2.921 ***	−4.085 ***	2.725 *
Major of study	−0.027	0.047	-0.181
Continent	2.364 *	1.774 *	3.780 ***
Living conditions	−0.077	−0.491	−0.896

Values with * indicate significance at *p* < 0.05. *** Indicate significance at *p* < 0.001.

**Table 5:** Perception of academic resilience among respondents (N = 383).

Academic Resilience Component	N = 383	
	*n*	%
Mental health (SR1)		
Low (<3.04)	101	(26.37)
Moderate (3.04–6.52)	205	(53.52)
High (>6.52)	77	(20.10)
Academics (SR2)		
Low (<2.62)	64	(16.71)
Moderate (2.62–5.95)	217	(56.66)
High (>5.95)	133	(26.63)
Relationships (SR3)		
Low (<10.80)	104	(27.15)
Moderate (10.80–14.55)	151	(39.43)
High (>14.55)	128	(33.42)

**Table 6:** Resilience scores across sociodemographic characteristics among respondents (N = 383).

Sociodemographic Characteristic	Mental Health (SR1)		Academics (SR2)		Relationships (SR3)	
	M (SD)	*p*-Value	M (SD)	*p*-Value	M (SD)	*p*-Value
Sex		0.041 ^1^		0.287 ^1^		0.806 ^1^
Male	5.07 (2.00)		4.39 (1.97)		12.78 (4.01)	
Female	4.63 (1.90)		4.23 (1.80)		12.62 (4.24)	
Marital status		0.024 ^1^		0.109 ^1^		0.045 ^1^
Single	4.83 (1.94)		4.25 (1.86)		12.57 (4.11)	
Non-single	3.78 (1.70)		5.00 (1.82)		14.83 (4.62)	
Year of study		0.009 ^1^		<0.001 ^1^		0.012 ^1^
Undergraduate	4.91 (1.92)		4.09 (1.76)		12.42 (4.00)	
Postgraduate	4.29 (1.99)		5.07 (2.06)		13.70 (4.63)	
Major of study		0.426 ^2^		0.027 ^2^		0.036 ^2^
Education	4.00 (1.49)		3.95 (2.11)		12.00 (3.48)	
Science/Medicine/Biology	4.75 (2.27)		4.98 (1.79)		14.48 (4.80)	
Engineering	4.88 (2.13)		5.06 (2.14)		13.69 (4.54)	
Agricultural	6.50 (0.71)		6.00 (1.41)		8.00 (0.00)	
Business	4.76 (1.91)		4.21 (1.82)		12.56 (4.08)	
Social science/Law	4.97 (1.90)		3.79 (1.54)		11.70 (3.61)	
Language/Arts/ Communication	4.92 (1.94)		4.21 (1.91)		12.52 (4.12)	
Continent		0.070 ^1^		0.258 ^1^		0.118 ^1^
Asia	4.69 (2.00)		4.23 (1.89)		12.86 (4.24)	
Non-Asia	5.00 (1.78)		4.44 (1.78)		12.21 (3.92)	
Living conditions		0.297 ^2^		<0.006 ^2^		0.001 ^2^
Alone	5.03 (2.06)		4.71 (1.92)		13.11 (4.27)	
With friends	4.59 (1.87)		4.13 (1.77)		12.92 (3.93)	
With relatives	4.73 (1.95)		3.62 (1.92)		10.70 (4.30)	
Others	4.96 (1.77)		4.22 (1.72)		11.48 (4.15)	

^1^ Mann–Whitney *U* test. ^2^ Kruskal–Wallis test.

**Table 7:** Multivariate analyses on sociodemographic characteristics with academic resilience.

Sociodemographic Characteristic	The Beta Coefficient (β)		
	Mental Health (SR1)	Academics (SR2)	Relationships (SR3)
Sex	−0.424 *	−0.036	−0.030
Marital status	−0.574	0.373	1.811 *
Year of study	0.038	−0.023	−0.093
Major of study	−0.493	0.862	0.799
Continent	0.260	0.261	−0.567
Living conditions	−0.053	−0.319 **	−0.785 **

Values with * indicate significance at *p* < 0.05. ** Indicate significance at *p* < 0.01.

**Table 8:** Correlation matrix of academic burnout and academic resilience by subscales among respondents (N = 383).

	EX	CY	EF	SR1	SR2	SR3
EX	1.000					
CY	0.749 ***	1.000				
EF	0.181 ***	0.070	1.000			
SR1	0.026	−0.024	0.014	1.000		
SR2	−0.064	−0.104 *	0.333 ***	−0.030	1.000	
SR3	0.006	−0.111 *	0.310 ***	−0.031	0.447 ***	1.000

Values with * indicate significance at *p* < 0.05. *** Indicate significance at *p* < 0.001. Note: EX = Exhaustion; CY = Cynicism; EF = Efficacy; SR1 = Mental Health; SR2 = Academics; SR3 = Relationships.

**Table 9:** Multivariable linear regression model of the relationship between academic resilience and academic burnout.

Sociodemographic Characteristic	The Beta Coefficient (β)		
	Exhaustion (EX)	Cynicism (CY)	Efficacy (EF)
Age	−0.250 *	−0.082	−0.091
Sex	0.420 *	0.837	−0.746
Marital status	1.705	0.219	−0.229
Year of study	−2.850	−3.278 **	2.127
Continent	1.705	1.809 *	3.887 **
Living conditions	−0.314	−0.709	−0.171
Financial status	−1.989 *	−0.552	0.399
Academic resilience			
Mental health	0.210	−0.073	0.010
Academics	−0.147	−0.013	1.181 ***
Relationships	0.064	−0.180	0.482 ***

Values with * indicate significance at *p* < 0.05. ** Indicate significance at *p* < 0.01. *** Indicate significance at *p* < 0.001.

## Data Availability

The data presented in this study are available on request from the corresponding author.
